# Gender and social protection and health policies promoted during the COVID-19 pandemic: Global scoping review and future challenges

**DOI:** 10.7189/jogh.12.05056

**Published:** 2022-12-29

**Authors:** Daniela Luz Moyano, María Lara Martínez, Laura Lara Martínez

**Affiliations:** 1 National University of La Matanza, La Matanza, Argentina; 2 National University of Cordoba, Cordoba, Argentina; 3 The Distance University of Madrid, Madrid, Spain

## Abstract

**Background:**

Governmental interventions have been important tools for mitigating COVID-19 transmission, but they have also negatively impacted different gender-related components. We aimed to answer the following questions: What is the scope of the gender approach in the literature analysing health and social protection policies promoted during the COVID-19 pandemic? What are the challenges and recommendations for gender-sensitive policies for the post-pandemic and future crises?

**Methods:**

The study design is based on three stages: a global synthesis of the evidence through a scoping review, the generation of a framework of emerging inequalities based on sociocultural markers, and the creation of a matrix with the challenges and recommendations. In this scoping review, we searched 10 online databases for studies published until April 2022 and conducted a content analysis on the extracted studies.

**Results:**

Of the 771 identified records, 67 met our inclusion criteria. Most studies had a female person (52/67) as the first author. The binary model was the main approach addressed in the studies (61/67). The literature showed that the closure, distancing, and other social policies did not include a gender approach and generated negative gaps related to economic instability, reproductive roles, and gender violence. In the intersectionality dimension, multiple aspects emerged (macro, meso, micro-social level, and individual level). Greater gender gaps in connection with employment (related to increased housework) were observed during the closure and distancing stage of the pandemic. Asymmetries related to female participation in the management of the pandemic and an increase in discrimination and abuse of diversity groups were detected.

**Conclusions:**

We observed gaps both in the gender approach both in knowledge and in policy implementation during the pandemic in the different countries explored in this work. This is a call to attention and action for researchers, political decision-makers, and other interested parties to incorporate and accentuate the gender perspective in all policies related to the post-pandemic period and future social and health crises.

Gender-related rights are a matter of public policy [1], which conversely plays a central role in gender equity. Government interventions have sought to mitigate the spread of COVID-19 but have not addressed the impacts on gender [2].

Recognizing the extent to which pandemics disproportionately affect gender is critical to understanding their primary and secondary effects [2]. The conception of gender assumes different cultural forms and meanings and is traversed by political, social, historical, and cultural issues, as well as mechanisms of domination and power [3,4], making it crucial when designing and implementing effective and equitable policies and interventions [2].

Prior studies showed a limited gender scope with variations in the degree of its inclusion during the development of health policies [5]. The forms of organizations of the health sector and these policies could be thought of as a form of “biopower” [6] where social phenomena are naturalized by being characterized as biological, contributing to and deepening gender inequalities.

An overlap between gender identity and the invisibility of work was observed at the domestic level [7], where care and domestic tasks are complemented by paid employment [8], especially during COVID-19 [7].

Lockdowns and school closures during the pandemic have had serious consequences on different gender-related dimensions of general well-being, with a significant increase in the burden of unpaid work, especially for women, who provide most of the informal care, limiting their work possibilities, their economic capabilities, and opportunities [2,9]. Intersectionality [9,10] plays a central role in the analysis of public policies. The intersections of various structures in places of power such as the public, the home, and the intimate space, make women ultra-vulnerable groups [9].

Patriarchal norms were amplified in the crisis, along with gender-blind policies, have made women “invisible” [9], as well as other diversity groups and populations exposed to multiple inequity factors [11].

As experience and literature to date have shown, the COVID-19 pandemic has generated multiple negative gender-related impacts in all areas, especially health and socio-economic conditions [12,13]. In this complex context, there is a need to establish urgent measures that incorporate a gender approach in preparedness and response [2], but also in recovery from the crisis.

We aimed to explore the following questions: 1) What is the scope of the gender approach in the literature analysing the health and social protection policies promoted during the COVID-19 pandemic? 2) What are the challenges and recommendations for gender-sensitive policies for the post-pandemic and future crises?

## METHODS

### Study design

We designed a study with three stages: 1) A global synthesis of the evidence through a scoping review [14,15], where we presented an overview of a diverse body of literature on the inclusion of the gender approach [3,4,10,16] focused on inequities in the health and social protection policies promoted during the COVID-19 pandemic; 2) The generation of an emergent framework composed of socio-cultural markers of gender inequities by conducting a content analysis of the literature; and 3) The creation of a matrix with the main challenges found in the literature and recommendations to be considered during the post-pandemic and future health crises.

### Outcomes and definitions

Based on the analysis of the gender approach [3,4,10,16], the following dimensions and sub-dimensions were defined:

#### Scope of gender in the literature

Context: the COVID-19 pandemic was an inclusion criterion. The categories were: geographic location (country), temporality (year of publication and period of analysis), type of study design (or methodological approach, in the case of not reporting the design), objective, first author’s gender (categories were imputed: male/female, based on prefixes, pronouns, names, and online bibliographies), type of main policy addressed, main characteristics of the policy/ies, and main reported results related to gender.

Theoretical-methodological approach to gender: According to a global analysis of available documents, the predominant gender approach was imputed, with two categories – binary and inclusive.

Use of language: Language conveys cultural and social attitudes [17]. Based on a global analysis of available documents, the following categories were imputed: female/male (terms included: female/male, woman/man, girl/boy, mother/father) or gender diverse (terms included: lesbian, gay, bisexual, transgender, travesty, intersex queer, other identities, and LGBTIQ+).

#### Socio-cultural markers of emerging gender inequalities

From the global content analysis of all documents, the following dimensions were defined from which the emerging socio-cultural markers (categories) emerged, from which an emergent framework was generated and challenges and recommendations identified.

Intersectionality: This is the recognition of the combined effects of the social categories of race, class, and gender [10]. Macro, meso, and micro-social levels were considered.

Gender roles: A set of culturally defined expectations that are assumed, learned, and performed according to gender [3].

Asymmetries and inequalities: Societies and cultures reproduce hierarchical structures articulating relations of power, domination, and subordination that translate into inequalities [3,4]. These inequalities should also be explored in public policymaking [16].

### Selection criteria

During the review stage, all texts published at the international level that met the following inclusion criteria were included “post hoc”, based on familiarization with the literature: texts that referred to the explicit, central, and transversal inclusion of the gender approach, with a focus on inequities in the context of health or social protection policies promoted during the COVID-19 pandemic. All documents were included without temporal, language, or geographic restrictions, with different designs: systematic reviews, narrative reviews, experimental, observational, descriptive studies, essays, viewpoints, qualitative studies, and policy briefs.

We excluded publications on policies implemented before the COVID-19 pandemic, that were not promoted by governments, that did not have sufficient information on a specific case of public policy (for example, those studies that were only limited to enunciating the name of a policy without a specific description), that did not explicitly report the incorporation of the gender approach, that were preprints, abstracts, presentations at scientific meetings, editorials, or research guidelines, and those whose full text could not be accessed. Opinion pieces, written press, political ads, official notices, and public presentations were not included, and neither were papers focusing only on research or policy “gaps”, reflections, recommendations, future perspectives, framework development, or lessons learned from health crises prior to COVID-19. Documents that were not captured by the database search or reference screening were not included.

We did not include studies that only reported population outcomes or impacts (deaths, cases, hospitalizations, health spending, mental health or other chronic diseases, unemployment indicators, loss of income, lower school performance, mental health, crisis recovery, vaccination, mobility patterns, violence date, individual experiences, intimate arrangements, scholarly productivity of women, etc.) and did not focus on policies in specific countries, or that specifically analysed system-level interventions, health services and personnel, violence victim care services, business or school contexts, or reported policy characteristics in a grouped, synthetic, or global manner from various countries. We excluded specific publications on fiscal policies, macroeconomics, data, online education, remote work or school, or other educative institution closure or business policies, implemented during the COVID-19 pandemic, and that were not directly addressed to the communities. Studies analysing any social or health policies in the context of a pandemic related only to specific groups (such as schoolchildren, college students, children, adolescents/youth, elderly people, patients with chronic diseases, and pregnant persons, women in rehabilitation, risk behaviours, postnatal women, or people with HIV) were not included.

We did not include studies analysing only leadership, political discourses, press conferences, or social networks information and media.

We did not include technical documents from government, multilateral agencies, and other organizations that were not captured by the initial search strategies in the database, or studies with potential conflicts of interest.

### Search strategy

The data collection was carried out from March 5 to April 4, 2022, and the search was updated on August 15 to 18, 2022 (only on some databases by a rapid review). Ten specialized scientific databases were reviewed from different areas (multidisciplinary, health sciences, public health, social sciences, humanities, and gender): ScienceDirect, MEDLINE (via PubMed), Google Scholar, Anthropological Index Online, JSTOR Journal Storage, SAGE Journals Online, Springer, Studies on Women & Gender Abstracts, Cochrane Library and Global Index Medicus (GIM) – which includes regional indexes (African Index Medicus (AIM), Latin America and the Caribbean Literature on Health Sciences (LILACS), Index Medicus for the Eastern Mediterranean Region (IMEMR), Index Medicus for South-East Asia Region (IMSEAR), Western Pacific Region Index Medicus (WPRO)), with an independent search in LILACS.

We generated specific search strategies for each database based on combined terms in English, Spanish and Portuguese. Before developing the strategies, we carried out an exhaustive analysis of the dimensions and scope of this study, making it possible to generate sensitive strategies that we tested and refined to capture the greatest number of documents of interest (Appendix S1 in the **Online Supplementary Document**).

We also used the Google search engine to manually retrieve the references of both included and excluded articles captured by the initial search strategy, for which we conducted a full-text review. We eliminated duplicates found in the different databases.

We first evaluated the study titles and abstracts, after which we analysed the full text. If the full text of potentially a relevant scientific article could not be located, we tried to contact the author. Citations were managed with Endnote.

We prepared a register form to systematize the information of each document, which contained the pre-established dimensions and categories of analysis, with an open field to record emerging information.

### Data extraction and analysis

We conducted a content analysis on the information gathered from the documents [18]. To improve internal validity and mitigate possible information bias, all the information collected was reviewed at least twice. Three independent reviewers (including DLM, MLM and LLM) assessed the included documents for relevance, detecting potential discrepancies and finally reaching a consensus on the final inclusion. We developed an emerging framework of socio-cultural markers and a matrix of challenges and recommendations.

### Ethical considerations

The study was based on published documents. The manuscript was evaluated by the Institutional Health Research Ethics Committee (CIEIS) of the Hospital Nacional de Clínicas of the National University of Cordoba, Argentina (Number: PV-2022-00490734-UNC-CE#HNC).

## RESULTS

### Scope of gender in the literature

**Figure 1:**
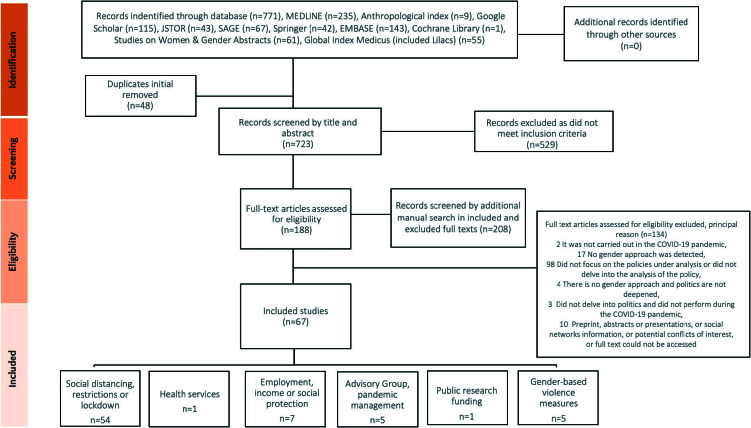
The scoping review flowchart.

A total of 771 records were retrieved, 188 of which underwent a full text analysis. 208 records were screened through the additional manual search described above (**Figure 1**).

A total of 67 articles were included (54 from the initial search strategy and screening and 13 from the citation screening (**Figure 1**)). Included articles written in other languages (Spanish = 3, German = 1, Vitimite = 1, Portugese = 5) were translated into English.

The included studies [19-85] either covered at least one country from the European region (n = 18), or countries from the Americas (n = 21, 13 from Latin America and 8 from North America), Africa (n = 10), Asia (n = 20), or Oceania (n = 4), while one had a global scope.

Fifty-four studies dealt with social distancing, restrictions, or lockdown policies, seven with employment, income, or social protection, and five with gender-based violence measures (**Table 1** and **Figure 1**). Most studies were conducted during 2020 (the first year of the pandemic caused by COVID-19) and had a female first author (n = 52/67) (**Table 1**).

**Table 1:** Description of the studies selected in the global scoping review according to the dimensions of analysis

Citation	Year of publication	Country	Type of policy addressed	Main reported characteristics of the policy	Type of design or methodological approach	Objective of the study	Main gender-related results reported	Year/period of analysis	Gender of 1st author	Gender approach	Use of language
Koch et al. [19]	2022	European countries	Government responses to COVID-19, containment, lockdown, health system, and economic policies (income, debt/contract relief)	Containment and closure policies, health system (such as investments in care, public information campaigns, contact tracing) and economic policy (financial support to individuals).	Multilevel model	To assess how government responses the relationship between gender and psychological distress.	Containment and closure and health system policies increase psychological distress more in women than in men.	2020	Male	Binary	Female/male
Kim et al. [20]	2020	South Korea	Pandemic response in quarantine phases and mitigation	Quarantine policy: screening, mass testing, monitoring, isolation in government centres, home quarantine, or in hospitals. Mitigation policy: social distancing and school closures.	Gendered lens	Analysing the experience during the COVID-19 outbreak through a gender lens.	Gender should be incorporated into all stages of response. Formal and informal care work should be considered essential. Prioritize a people- and women-centred approach. Technologies and resources should be distributed equitably.	2020	Female	Binary	Female/male
Omukuti et al. [21]	2021	Latin America and Sub-Saharan Africa	Response to COVID-19 from international, regional, and national governments	National responses: focus on the infected, and border closures. Financial dependence, reduction of public spending on social services.	Viewpoint documentary analysis, interviews	Reflections on the responses to COVID-19 from international and regional organizations and national governments.	The national policy responses have been partial and inconsistent with respect to gender in Sub-Saharan Africa and Latin America without recognizing the multiple drivers of inequalities.	2020-2021	Female	Binary	Female/male
Rieger et al. [22]	2022	United States	Social distancing policies	Local and state governments issued distancing and shelter-in-place policies. With recommendations to stay at home, distancing, school closing.	Social-ecological model, intersectional lens, and recommendations	To explore how the risk of GBV has been exacerbated during the current pandemic.	A comprehensive framework that adapts an intersectional lens to explain changes in risk and protective factors for GBV. It highlights the differential impacts of social locations (race, gender, social class).	During the current pandemic	Female	Binary	Female/male
van Daalen et al. [23]	2020	Worldwide	National working groups to prevent, monitor and mitigate COVID-19	Decision-making through traditional processes: directors of ministries, experts, and heads of well-known institutions.	Quantitative data	Assessing the gender gap in COVID-19 working groups.	Most groups tend to be occupied by males, with only 3.5% exhibiting gender parity. Males were overrepresented in global task forces.	2020	Female	Binary	Female/male
Witteman et al. [24]	2021	Canada	Gender policy in COVID-19 public research funding	A national health research funder implemented gender policy changes. Included extension of deadlines, and consideration of sex and gender in grant requirements.	Data analysis before and after	To compare the application and success rates of grants submitted by researchers with different identity characteristics.	After the policy changes, the funder received more applications from females and awarded a greater proportion of grants compared to males. Received and funded more applications that considered sex and gender in research.	2020	Female	Binary	Female/male
Wenham et al. [25]	2021	United Kingdom (UK)	COVID-19 SAGE	SAGE is a group of experts advising the government on scientific and technical aspects of emergency response and recovery.	Policy analysis	To understand whether and how gender had considered by the SAGE.	Explicit references to females were largely of a biological nature. The references to the gender impacts of policies largely reproduced gendered stereotypes and roles.	2020	Female	Binary	Female/male
Cook et al. [26]	2021	Germany, Italy, Norway, United Kingdom (UK)	Social and employment policies established during the crisis	Interventions have focused on business support, job retention, workplace safety, and preventing social hardship.	Comparative analysis	Gendered analysis of the design, access, and impacts of COVID-19 in social and employment policies.	Countries have implemented some form of short-time work scheme to support income. The schemes largely assume a normative (male) worker and leave unchallenged the gender division of domestic work.	2020	Female	Binary	Female/male
Ruiz-Pérez et al. [27]	2021	Spain and autonomous communities	Government containment measures and contingency initiatives against GBV	Decree-Law to assist victims of gender violence in the crisis. In the autonomous communities, programs and measures were adapted to assist female victims of GBV	Review	Review of the measures adopted by the government to prevent GBV.	In economic uncertainty, it is not possible to prevent GBV, without considering the increase in unemployment, job instability, economic dependence or the overload of reproductive tasks.	So far	Female	Binary	Female/male
Sulistyawan et. al. [28]	2020	Indonesia	Governmental regulation of restrictions on the handling of COVID-19	The government issued a policy of large-scale restrictions through the issuance of several protocol guidelines.	Qualitative method, socio-legal approach	Analysing gender justice in COVID-19 management policy.	This policy poses a wide range of impacts on females in the context of the family and as a female worker.	2020	Male	Binary	Female/male
Bacigalupe et al. [29]	2022	Spain	Expert committees for management and policy decision-making during the COVID-19 pandemic	Committees have been highlighted as important tool for crisis management and policy decisions.	Peer review	Analyse the gender composition of expert committees for management and policy decision-making during COVID-19.	75% of all committees represented females below the parity threshold.	2021	Female	Binary	Female/male
Blofield et al. [30]	2021	Latin America, Middle East, and Africa	Government policies on treating GBV during COVID-19	Tools to provide violence services were based on outreach campaigns, hotlines, first response health, legal and social services for survivors, and shelters.	Assessment of policies	Policy reviews, assessed the government's efforts to address GBV since the pandemic.	Despite the diverse cultural and political contexts there were challenges faced by people at risk of violence in confinement such as contacting emergency services, medical, legal and social assistance and shelters.	2020	Female	Binary	Female/male
Chitando [31]	2020	Zimbabwe and South Africa	Governmental responses to COVID-19, lockdown	Physical distance was one of the governments’ responses to COVID-19.	Literature search	The implications of government responses on the livelihoods of women.	Women and girls have been rendered vulnerable due to the methods adopted by governments in curtailing the spread of the coronavirus. Need to reduce the gender gap in public health policies.	Does not inform	Female	Binary	Female/male
Kampire [32]	2021	Uganda	Policy and legislation on GBV before and after the first wave of COVID-19	In presidential statements during the lockdown and quarantine, did not emphasize GBV or child abuse as priority areas.	Policy brief	Describe the impact of COVID-19 measures on violence against women and children services, programs, and policies.	The measures implemented to control the spread of COVID-19 increased the risks of violence against women and children and limited the ability of survivors to distance themselves from their abusers.	1995-2021	Female	Binary	Female/male
Oladeinde [33]	2021	Nigeria	Lockdown and subsequent relaxation	The first immediate response has been to introduce protocols, and lockdowns, with subsequent “relaxation”.	Gender lens	Evaluate the to which confinement affected the socio-economic activities and living conditions of people in the informal sector (particularly the women).	Policy measures remain inadequate and unsustainable to lift people out of the cycle of poverty. With a weak health system and a deficient institutional framework to manage the pandemic.	Does not inform	Male	Binary	Female/male
Rodriguez Fernandez [34]	2022	Spain	Measures to contain GBV during confinement	Public responses to GBV in the pandemic and initiatives of non-governmental organizations.	Systematic bibliographic review	Review of measures to prevent GBV and an analysis of the gender impact during the pandemic.	The crisis will have serious consequences for females by increasing social and labour inequalities, and greater contagion by occupying feminized employment. Confinement with the aggressor is a determining factor.	First months of confinement	Female	Binary	Female/male
Soremi et al. [35]	2021	Canada and Scotland	Public Health Policies to Address the COVID-19 Pandemic (lockdown)	The effects of the pandemic spurred many leaders to swiftly initiate policy measures to contain the spread.	Analysis of the case studies, document analysis	To analyse policy response to the pandemic and influence of gender in the solutions.	Solutions aimed at curbing COVID-19, did not conform to gender expectations. Policies by female leaders in times of uncertainty will not necessarily focus on or rely on gender considerations for legitimacy.	Between the moment when COVID-19 received political attention and declaration of lockdown	Female	Binary	Female/male
Perez-Brumer et al. [36]	2020	Peru	Social distancing, mobility restrictions	Legislated policies to enforce physical distancing by restricting mobility based on binary gender understandings and associated norms.	Does not inform	Assessment of the Peruvian case about their restricting the mobility based on gender.	COVID-19 and the gender-based policy have magnified violence and marginalization of transgender communities at the hands of the police.	2020	Female	Inclusive	Gender diverse
Foley et al. [37]	2021	Australia	Policy Responses to COVID-19 on Employment	Early access to retirement, industry-focused economic stimulus measures and flexible work arrangements.	Review	Explore the economic and social impact of the pandemic on women workers.	Women workers were disproportionately affected by the pandemic and policy responses have largely failed to recognize and redress the gendered impacts of the crisis.	2020	Female	Binary	Female/male
Bariola et al. [38]	2021	Denmark, Germany, and the United States	Policy responses for the pandemic	The welfare infrastructures of corporatist states like Germany and social democratic states like Denmark and liberal states like the United States.	Feminist approach to welfare states	To examine response policies in three countries with different welfare state regimes.	Policy responses in all three countries are shaped by cultural frameworks about state-market-family relations and the role of the state in the economy in general and during economic crises.	2020	Male	Binary	Female/male
Polischuk et al. [39]	2020	Argentina	Multilevel government response to GBV during the first month of “stay-at- home”	National and provincial governments enacted responses to gender-based violence that targeted systemic causes of gender-based violence, continuity of existing services, and generated new communication strategies.	Viewpoint essay	To describe the government's response to address GBV during the first month of the “stay-at-home” order.	Continuity of existing services and new communication strategies during the pandemic. Government organizations should consider the gendered effects of responses and respond through a multi-level, cross-sectoral response.	2020	Female	Binary	Female/male
Arora et al. [40]	2021	India	Complete lockdown	For the lockdown, migrant workers like those employed as domestic helps, among others were forced into an indefinite nonpaid leave or asked to leave with one month’s wage.	Narrative study	To understand the lived experiences of migrant women workers during the pandemic and of gendered inequality.	The pandemic exacerbated the already precarious positions of the women by creating a situation where: patriarchal structures were further reinforced, losing gender solidarity and companionship through social distancing.	2020	Female	Binary	Female/male
Craig et al. [41]	2021	Australia	Social distancing, closures, lockdowns, supported with government income maintenance	A wage subsidy program was implemented, paying employers for each employee they kept on the payroll, and adjusted the childcare subsidy system.	Online survey	To report early results on how the pandemic affected paid work, domestic work, and caring responsibilities.	Women shouldered most of the extra unpaid workload, but men’s childcare time increased more in relative terms.	2020	Female	Binary	Female/male
Holder et al. [42]	2021	United States	The shelter-in-place orders, mandatory shutdowns of nonessential businesses	Aggregate demand for many goods and services halted, while many suppliers were mandated to temporarily close.	Quantitative analysis	Investigate the occupations in which black women experienced the greatest job losses during the early phase of the pandemic.	The greatest losses were cashier jobs in the hotel and restaurant industry, and childcare worker positions in the health care and social services industry. These two occupations are low wage and dominated by women.	2020	Female	Binary	Female/male
John et al. [43]	2021	Kenya	Restrictive policies	Government-imposed restrictions (quarantines and closures) increase exposure of women and girls to gender based violence (GBV), and further heightened due to diminished access to GBV services.	In-depth interviews	Understand how COVID-19 containment polices were impacting women and girls, as well as availability and access services.	The COVID-19 containment measures heightened the exposure of women and girls, particularly those harder to reach, to forms of GBV.	COVID-19 restrictions in place	Female	Binary	Female/male
Singh et al. [44]	2022	India	Strict lockdown	India's countrywide lockdown was ranked as one of the severest across of the world.	Qualitative research	Experiences of women in informal employment in Indian Punjab during the COVID-19 crisis.	Containment measures and the stringent lockdown imposed to control the spread of COVID-19 exposed existing vulnerabilities, reinforced economic inequities.	2020	Female	Binary	Female/male
Chakraborty [45]	2021	India	Lockdown	COVID-19 pandemic and the lockdown have impacted in the political, structural, economic systems and engendered the growing rifts.	Framework of biopolitics and necropolitics	To study the ways in which the pandemic and the lockdown prompted political, social, and economic precarity and how such predicaments affected the lives of the marginalized, in particular, of women.	State and the society following the lockdown and impending economic crisis stigmatizes, injures, and even eliminates women (from different social strata).	During the pandemic crisis	Female	Binary	Female/male
Desai et al. [46]	2021	India	Lockdown	India implemented one of the world’s most stringent lockdowns in response to the COVID-19 crisis.	Logistic regression models	Examine whether the impacts of the lockdown on employment differed by gender in Delhi.	Women experienced greater job losses than men with predicted probability of employment declining by 72% for women and 40% for men.	2019-2020	Female	Binary	Female/male
Chauhan [47]	2021	India	Lockdown	The government imposed a nationwide lockdown.	Qualitative approach	To study the impact of COVID-19 on unpaid work and gender differences in the urban centres in India.	Women were already sharing a higher burden of unpaid work, and COVID-19 and the lockdown has worked to increase their burden of unpaid work even more.	During lockdown and social distancing	Female	Binary	Female/male
Sell et al. [48]	2021	German	Expert committees	Large numbers of decision-makers turn to scientific experts to obtain information and to legitimize or justify decisions with the inclusion of scientific expertise.	Multi-stage document analysis	Examine the expert committee’s advisers the governments, in disciplinary composition, gender representation and transparency.	In eleven committees, the members were known by name, women making up 26% of the members. The members of ten committees were not known.	2020	Female	Binary	Female/male
Santana et al. [49]	2021	Brazil	Social distancing	Officials and health authorities have encouraged the adoption of protective measures, with an emphasis on social distancing through quarantines.	Historical aspects and recommendations	To reflect about the social impacts of pandemic, and the strategies to deal it on the everyday life of LGBTI+.	It was observed how the pandemic strongly impacted in LGBTI+ population. Its consequences are immeasurable and deepen the inequities and social processes intertwined with the LGBTI-phobia.	Does not inform	Male	Inclusive	Gender diverse
Corrêa et al. [50]	2021	Brazil	Emergency assistance and social distancing	In the pandemic, there was difficulty in obtaining food parcels or poor quality, and the slow payment of the Emergency Aid.	Qualitative approach	Discuss of the forms of violence experienced at the intersection of race, gender and class in a community of social vulnerability during the pandemic and before it.	During the pandemic and before it, to structural violence linked to race, class and gender, expressed in the inaccessibility to decent conditions of housing, food and basic income.	2017-2020	Female	Binary	Female/male
Camilo et al. [51]	2021	Brazil	Restrictions	Restriction of care by the single health system, termination of Emergency Aid. Reducing reception spaces for victims of child and gender violence.	Qualitative research	To understand the facets of care in a territory of social exclusion affected by the coloniality kept in force by neoliberal capitalism.	The challenges encountered stem from the complete social exclusion (black women are the most affected) without basic rights and abandoned by public authorities, in food and housing insecurity. Pandemic aggravated the situation: domestic violence, femicide, paedophilia, mental illness, and suicides.	2017-2020	Female	Binary	Female/male
Irons [52]	2022	Peru	Gender-segregated quarantine	During first lockdown, one was carried out a gender-segregated quarantine policy drove women to the marketplaces in masse on so-called ‘women’s days.	Intersectional, post-colonial framework	To address the social and cultural structures that explain why and how gender-segregated quarantine a policy could have been implemented.	There were cases in which gender was foregrounded by pandemic policy with limited success, such as the gender-segregated quarantines. Though, gender has indeed been widely omitted from central pandemic responses.	2020	Female	Binary	Female/male
Meegaswatta [53]	2021	Sri Lanka	Lockdown and social distancing	The government imposing a full lockdown, the ‘curfew’, was police-managed with a zero-tolerance policy.	Structured online questionnaire	Explore how formally employed women navigated the COVID-19 lockdown.	Majority of women worked from home, had to balance their professional and personal commitments in a time of increased uncertainty and anxiety.	2020	Female	Binary	Female/male
Shafer et al. [54]	2020	Canada	Lockdown	Closing of businesses, workplaces, schools, and daycare centres, prohibition on gatherings.	Qualtrics online panel	Perception of task sharing and increased participation of fathers in housework and childcare both before and during the pandemic.	Small shifts toward a more equal division of labour in the early lockdown months, with increased participation in housework and childcare by fathers.	2020	Male	Binary	Female/male
Bau et al. [55]	2022	India	Containment policies	While India initially pursued a nation-wide lockdown, from June 2020, it had a patchwork of containment zones in which lockdown measures were imposed.	Phone survey	Estimate association between the pandemic and containment policies and measures of women’s well-being (mental health and food security).	The greater prevalence of containment policies is associated with increased food insecurity and reduced mental health in women.	2020	Female	Binary	Female/male
Santos et al. [56]	2020	Brazil	Quarantine	At the beginning of the quarantine, the banking institution did not accept the refugee document. During the quarantine period, the low-cost social restaurant closed.	Data and information from official websites, and narrative	Perspectives on the refugee and migrant LGBT population in the context of the COVID-19 pandemic, in Manaus.	The LGBT population, transgender women, refugees and migrants are even more vulnerable during the quarantine. In the context of COVID-19, they remain helpless.	During quarantine	Male	Inclusive	Gender diverse
Srivatsa [57]	2020	Sierra Leone	Lockdown	Implemented a nationwide partial lockdown and curfew measures are in place.	Briefing note	Examine the impact of COVID-19 and policy responses on gender norms.	Before the onset of the pandemic, women held a weaker position in society (health, education, formal labour market, domestic violence, access to finance). Their bargaining power to obtain better solutions has been undermined by the pandemic.	Does not inform	Female	Binary	Female/male
Speed et al. [58]	2020	United Kingdom (UK)	Lockdown	Closure of non-essential businesses and venues, prohibition of all public gatherings and requiring everyone to stay at home.	Online questionnaire	Analysed the impact of Covid-19 on the ability of victims of GBV to access justice.	While GBV services have been able to demonstrate resilience in the weeks following the lockdown, many of the services are facing tough times ahead, because loss of funding; resourcing difficulties in moving services online, delivering services in a safe and effective manner.	2020	Female	Binary	Female/male
Cellini et al. [59]	2021	Italy, Belgium	Lockdown	Both countries established total lockdown. During the lockdown, most activities were closed.	Online survey	To investigate how the period of confinement affected self-reported sleep characteristics, with special regard to sleep timing and subjective quality.	In the lockdown, sleep quality was markedly impaired in both countries. The most vulnerable individuals appeared to be women.	2020	Male	Binary	Female/male
Walters et al. [60]	2022	South Africa	Lockdown	In the government-enforced lockdown, all non-essential businesses, schools, and universities, academics were constrained to work from their homes.	Mixed methods	To shed light on the complex reasons for the decline in research during the pandemic-enforced lockdown among women academics.	A dramatic increase in teaching and administrative workloads, and the traditional family roles assumed by women while “working from home”.	2020	Female	Binary	Female/male
Marques et al. [61]	2021	Brazil	Physical distancing	Brazil has a context of public governance characterized by the neoliberal aegis, the denial of scientific evidence in political decision-making, the dissemination of false news.	Critical essay	To addresses the interconnection between social markers of the inequalities that affect marginalized groups as sex workers, domestic workers and the LGBTQIA+ community.	The pandemic has exacerbated the processes of stigmatization, marginalization and exclusion in certain groups that were already suffering from it.	Does not inform	Female	Inclusive	Gender diverse
Jaim [62]	2021	Bangladesh	Complete lockdown	All the public and private offices were closed, the exception was applied to certain sectors (kitchen market and drug stores).	Qualitative method	To explore on women's experiences regarding their small businesses in a patriarchal nation during COVID-19.	Discloses patriarchal practices in relation to the discontinuation or closure woman businesses because of the pandemic.	Social distancing and the restricted mobility	Female	Binary	Female/male
Piscopo et al. [63]	2021	Brazil, United States, Philippines, Japan, Mexico, India	Pandemic management	From shutting down to expanding hospital staff, fell to lower levels of government.	Case studies	Analysed how women governors, mayors, and local officials promoted public health and social protection in countries where men chief executives failed to take steps to containment.	The women leaders prioritized clear communication and swift action and implemented policies that attended to the needs of vulnerable community.	From early 2020 through 2021	Female	Binary	Female/male
Seiz [64]	2021	Spain	Lockdown	Strict lockdown measures, whit the closure of schools and the interruption of most nonessential services.	Online survey	Analyses the intrahousehold division of labour within heterosexual couples with children during the lockdown.	A non-negligible proportion of the families exhibit traditional domestic work patterns, which highlights of normative structures binding women to the household sphere.	2020	Female	Binary	Female/male
Woskie et al. [65]	2021	Panama	Sex-Segregated Social Distancing Policy	Instituted a sex-segregated mobility policy to limit people’s circulation.	Retrospective analysis, Global Positioning System data	Looks at relative mobility for women and men, examining differences by volume and type of movement.	Identify lower visits to all community location categories on women-mobility days.	2020	Female	Binary	Female/male
Christl et al. [66]	2022	Austria	Discretionary policy measures	The short-time work (STW) scheme (a net replacement rate of up to 90%). Also, a one-off payment for the unemployed and Special payment for families (whit children).	Microsimulation	Analysed the impact of the pandemic on household income.	Females tend to experience a greater loss in terms of market income. Males profit mainly from short-time work scheme, while females profit from other discretionary policy measures, such as the one-off payment for children.	2020	Male	Binary	Female/male
Septarini et al. [67]	2021	Indonesia	Control measures	The control measures include stay-at-home orders, physical distancing, wearing facemasks, and regular hand washing.	Cross-sectional survey	Examines the psychological distress and happiness of men who have sex with men and transgender people in Bali during the pandemic.	The majority reported moderate to very high psychological distress during the pandemic. The factors to be significantly associated with higher psychological distress: being a student, higher levels of stigma, had ever experienced discrimination, felt better prior to the pandemic, and less happy than the average person.	2020	Female	Inclusive	Gender diverse
Yoosefi Lebni et al. [68]	2021	Iran	Quarantine	The countries have adopted quarantine as a means of preventing COVID-19 transmission.	Qualitative methodology	Describe the challenges of housewives during the COVID-19 quarantine.	During the quarantine, individual problems (low mental health and health), family (violence, economic situation, intensification of domestic tasks and roles) and social problems were identified.	2020	Male	Binary	Female/male
Pinchoff et al. [69]	2021	Kenya	Initial COVID-19 response, mitigation policies	Closure of schools, restaurants, and the prohibition of large gatherings.	Prospective cohort study	Assess the economic, social and health-related harm disproportionately affecting women of informal settlements, Nairobi.	More women than men reported adverse effects of mitigation policies. Women were 6 percentage points more likely to skip a meal with respect to men, and 8 percentage points more likely to report increased risk of household violence.	2020	Female	Binary	Female/male
Dasgupta et al. [70]	2021	India	Lockdown, social distancing	The stringent nationwide lockdown with curfew-like restrictions.	Interviews and observations	Investigates the impact of COVID-19 on the health and experiences of the transgender community.	Pandemic and the government strategies have exacerbated the challenges faced by the transgender community and threatened their survival.	2020	Female	Inclusive	Gender diverse
Doley et al. [71]	2021	India	Disruptions	The economic lockdowns were sudden and widespread.	Unit-level data sets, telephonic conversation, and informal interviews	Explores the impact of COVID-19 on unpaid work activities among women in the northeast states.	A more significant proportion of women than men were being employed in informal sectors and jobs in sectors that require close human interactions. With the enforcement of social distancing and lockdown, these sectors suffered more than formal sectors.	2020	Male	Binary	Female/male
Fuller et al. [72]	2021	Canada	Economic and social disruptions	Public health orders shuttered nonessential businesses.	Cross-sectional microdata	Document trends in gender gaps in employment and work hours over the pandemic.	During the pandemic, the gender gaps were larger among parents than people without children, and most pronounced when care and employment were more difficult to reconcile. When employment barriers eased, so did the gender–employment gap.	2020	Female	Binary	Female/male
Gordon-Bouvier [73]	2021	United Kingdom (UK)	Response to the pandemic	The forced closure of facilities and services.	Vulnerability lens	Critically analysed the UK’s response to the pandemic.	The pandemic has exacerbated harms and pressures for those performing paid and unpaid social reproduction. The response has retained a commitment to the autonomous liberal ideal, consequently ineffective.	To date	Female	Binary	Female/male
Jenkins et al. [74]	2021	Australia	Lockdown	State and Federal governments united to declare a lockdown, with plans to help businesses.	Gender perspective, invisibility of care work	Rethinking investment and infrastructure priorities for recovery in the light of a gender perspective on lockdown.	During the lockdown, acting as if home is a cost-less resource for appropriation, ignoring how home functions as a site of gendered relations of care and labour, shaped the invisibility of the imposition.	Recent experience of lockdown	Female	Binary	Female/male
Mahmood et al. [75]	2022	Bangladesh	Lockdown	Adopted different lockdown strategies. Public and private sector organizations remained closed (except for emergency services).	Secondary research	To analyse the impact of lockdown on intimate partner violence.	Decrease in the incidence rate of intimate partner violence-related calls during the pandemic, suggesting non-reporting of might have exacerbated during lockdown.	2019-2020	Female	Binary	Female/male
Uzobo et al. [76]	2021	African countries	Lockdown	The lockdown was declared in by most countries in Africa. Some of the countries started their lockdown with selected cities; others a nation-wide lockdown.	literature search	To explore domestic violence (DV) cases during the COVID-19 lockdown.	The lockdown worsens the already existing cases of DV. The response of the government has been very poor in terms of dealing with DV cases in the lockdown.	2010-2020	Male	Binary	Female/male
Yaish et al. [77]	2021	Israel	Economic lockdown	Was the first country to lock down the economy and tight quarantine.	Longitudinal survey	Discuss the implications of the lockdown on the balance among paid work, housework, and care work for men and for women.	As demand for housework by the lockdown increases, women, especial with children, increase their housework much more than men do, particularly when they work from home.	Week of March (before the first lockdown) until the second lockdown.	Male	Binary	Female/male
Hien et al. [78]	2021	Vietnam	Direct cash transfer and social protection policies for ethnic minority groups	Government policies focus on support to the most affected groups, that ethnic minority people, business households and enterprises in ethnic minority and mountainous areas.	Policy discussion	Examining the impact of pandemic on ethnic minority groups, especially women.	The pandemic has exacerbated difficulties within the ethnic minority and mountainous areas across from food security, livelihood, labour-employment, income and regular health care, education, and cultural and social affairs.	2017-2020	Male	Binary	Female/male
Craig et al. [79]	2021	Australia	Lockdown	Aggressive government response to COVID-19, national border closures, and workplace lockdowns, with government income maintenance.	National survey	Investigate how gender differences in time spent on paid and unpaid labour changed due to the pandemic.	Paid work time was slightly lower and unpaid work time was very much higher during lockdown than before it, were most for mothers. Though, the gender gaps somewhat narrowed.	2020	Female	Binary	Female/male
Del Boca et al. [80]	2020	Italia	Strict lockdown	Government imposed drastic measures to contain the growing epidemic (lockdown, regulations of the movement by individuals, school closures, and social distancing).	Survey data	Analyse the effects of working arrangements due to COVID-19 on housework, childcare and home schooling among couples.	Except for those continuing to work at their usual place of work, all of the women spend more time on housework than before, and do not seem to depend on their partners’ working arrangements.	2020	Female	Binary	Female/male
Borah Hazarika et al. [81]	2021	India	Lockdown	A nationwide lockdown, in order to ensure social distancing.	Interviews, secondary literature	The experiences of men and women (parents) in sharing household responsibilities and its impact on their careers during the lockdown.	The fathers and mothers were overworked and overburdened during the lockdown. For most mothers, their careers took a back-seat due to an increase in household chores and child-care.	2020	Female	Binary	Female/male
Sevilla et al. [82]	2020	United Kingdom	Social-distancing, lockdown	Self-isolation measures for those with symptoms were imposed, social-distancing measures, school and nursery closures and a general lockdown.	Online interviews	To document the impact of measures to control COVID-19 on families with children under the age of 12.	The families have been doing the equivalent of a working week in childcare, the mothers bearing most of the burden.	2020	Female	Binary	Female/male
Xue et al. [83]	2021	United Kingdom (UK)	Lockdown	Childcare facilities and schools were shut down, whit work from home. This nationwide lockdown signified severe restrictions on social contact.	Short web-survey	To describe how men and women divided childcare and housework during the lockdown, and whether were associated with worsening mental health.	Women spent more time on unpaid care work than men, were more likely to report increased levels of psychological distress.	2020	Female	Binary	Female/male
Zamberlan et al. [84]	2021	United Kingdom (UK)	Lockdown	Childcare and schools were closed. Social distancing, ‘stay-at-home’ measures and closures of public places and non-essential shops.	Web survey	Examines the impact of changes in paid working hours and time devoted to housework and childcare.	Both men and women who lost paid hours increased the time devoted to domestic chores. But, unpaid labour, particularly housework, remains a female responsibility in all the scenarios addressed.	2020	Female	Binary	Female/male
van Tienoven et al. [85]	2021	Belgium	Lockdown	Measures included the closure of schools, and non-essential shops. Limitation on events, mandatory telework, reduction of leisure activities, and closure of borders for non-essential travel.	Time-use survey	Analyses how gendered time-use and shares of routine and non-routine household labour changed under the influence of the COVID-19 pandemic and the lockdown.	The gender gap closes in absolute time, not in relative shares of routine and non-routine household labour.	2013-2020	Male	Binary	Female/male

GBV – gender-based violence, SAGE – Strategic Advisory Group on Emergencies, LBGTIQ+ – lesbian, gay, bisexual, trans, intersex, and queer+, LGBTIQA+ – lesbian, gay, bisexual, trans, intersex, queer, asexual+

Most studies approached gender through the binary model (61/67) with a predominantly male/female use of language, while some recognized that gender encompasses more than the binary and included other gender identities (eg, van Daalen et al. [23] and Cook et al. [26]).

These studies also recognized that the crisis is possibly affecting diverse gender identities [26] and that there is a need for intersectional data collection beyond a binary view [23] (**Table 1**). We found a gender-diverse approach in only six papers whose analysis focused on people of different gender identities [36,49,56,61,67,70]. We also found studies that only superficially mentioned LGBTIQ+ groups [22,34,39,50,52,54,58,65].

### Socio-cultural markers of emerging gender inequalities

**Figure 2:**
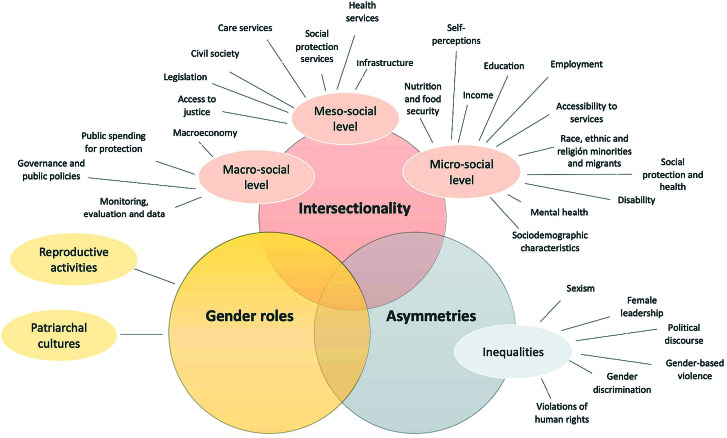
Emerging framework of socio-cultural markers of gender inequalities.

Most studies highlighted the absence of a gender approach in the government policies promoted during the pandemic (**Table 1**) by pointing out its implications on multiple socio-cultural markers related to gender inequalities **(****Figure 2**).

#### Intersectionality

Within the dimension of intersectionality, all studies have stressed or enunciated in their analysis at least some category at the macro, meso, and micro-social level (**Figure 2** and **Table 2**) [19-85]. From a micro-social sphere, markers related to mental health, sociodemographics, vulnerability conditions, food insecurity, loss and instability of employment, and loss of housing related to the pandemic, as well as policies taken by governments were observed. Employment and income policies had a central analysis in the works of Cook et al. [26], Foley et al. [37], Bariola et al. [38], Holder et al. [42], Fuller et al [72], Yaish et al. [77], and Hien et al. [78]. although in all the analysed studies, these markers were stated in some way during lockdown (eg, increased workload – formal, informal, domestic, and/or community work, loss or modality change of employment and income, feminized front-line employment and in other critical areas, sex workers in vulnerable situations, female-headed households, paid work and domestic work division, etc.) [19-85].

**Table 2:** Socio-cultural markers of emerging gender inequalities, challenges detected during the COVID-19 pandemic, and recommendations

Dimension	Socio-cultural markers	Challenges	Recommendations
Intersectionality	Micro-social level		
	Sociodemographic characteristics	Large families, informal urban settlements, rural residences, distance from central areas, lack of basic sanitation, vulnerable groups (elderly people, children, pregnant women, people with disabilities).	Work on barriers to access to health services, social protection, childcare and justice (physical, geographic, language, cultural, perceived, resources and, among others) seeking an adaptation to the specific needs of groups. Improve working conditions, especially for women and other vulnerable groups. Consider paid family leave, for maternity and paternity, and female empowerment with an active role of the unions. Generate income equity policies that promote security, protection, decent wages, professional progression, and economic autonomy. Promote legislation on pay equity and the near-term, policies should continue financial and protection assistance, principally in vulnerable groups. Promote the expansion of protection programs such as nutrition and childcare, and increased unemployment insurance benefits, considering the particularities and indicators in specific groups. Generate labour policies and job creation programs that include informal and temporary workers (mainly feminized), addressing aspects of labour quality and training. Women and others who assume care roles will require flexible work schedules with the presence of care policies (such as childcare and early childhood services). The homeworking can generate financial costs (electricity, telephone, and office equipment), that should be considered from employers. Investments in highly feminized labour sectors, this will lead to the creation of more jobs. It is important to consider gender disparity in industries when designing employment policies, for example, promoting the role of women in traditionally male occupations. Rebuild the participation of women and other vulnerable groups in the labour force and promote labour equity policies, and prevention of discrimination in search of a reduction in the wage gap. Help the reintegration into work of women and other groups of diversity affected by the crisis. Universal access to food assistance programs must be a priority during the crisis. Review its proper functioning and generate work between different sectors. Developing loans policy by according the specific problems of women in small businesses, especially in highly patriarchal countries.
	Mental health	Panic, anxiety, anger, stress, depression, uncertainty, stigma, discrimination, substance abuse, pre-existing mental health problems, grief, mental health may worsen in survivors of violence, suicides, fewer hours of sleep, mental health and services are stigmatized.	
	Disability	Difficulty with mobility and access to rehabilitation services.	
	Social protection and health, education	Feminization of poverty, loss of means of subsistence, housing problems and evictions, risk of sexually transmitted diseases and unwanted pregnancies, maternal and infant mortality, households headed by women, difficulty in accessing basic goods (hygiene, clothing, foods), low digital literacy, sexual education, access education, and autonomy in mobility, differences in levels of resilience.	
	Race, ethnic and religion minorities and migrants	Less access to employment, dependency on extended networks, inadequate care, illegal and legal migration, high concentration of refugee camps and shelters (with higher demand, spaces in poor condition), black women with disproportionately lost jobs, migrant or religion minorities job loss, social class and castes, colonial history, mass exodus of migrant workers.	
	Accessibility to services	Difficult geographic access (jobs, care services, courts), because of lack of public transportation, mobility restrictions and difficulty in access (interruptions and confinement) to social support, resources and essential health services (maternal and sexual health), childcare, justice services, care services for victims of violence, and medications (for example, antiretroviral or contraceptives).	
	Employment	Informality, sex work, precariousness, unemployment, exploitation, insufficient training, companies and jobs in the feminized sector were more affected, more women have lost their jobs (than men), lack of access to the employment support scheme, informal workers benefit disproportionately from protective measures, limited ability to work from home, layoff prevention package may have affected mothers (in a male breadwinner/female caregiver model), women out of the labour (withdrawal from free child care in the recessionary environment).	
	Income	Loss of income, wage gaps, weak financial safety nets, feminized low wages, economic dependency, limited savings, lack of coping strategies for economic survival, economic dependency and abuse, debt-default risks and imprisonment.	
	Self-perceptions	Fear of attending health services, limited awareness of rights and protection laws, and stigmatized perception of well-being.	
	Nutrition and food security	Food shortages with rising prices, risk of malnutrition (due to micronutrient deficiencies and the presence of chronic diseases) and food insecurity due to cultural norms (women and girls sacrificing their food consumption for others in their family), situations of hunger in poor woman and in victims of violence, limited government food distribution scheme (during lockdown), with bureaucracy, lack of complementary measures (such as free transport), without establishing mechanisms to ensure that vulnerable families access food through different means (for example, NGOs), insufficient food baskets, or poor quality, closure of low-cost restaurants, affected food supply chains (with lack of incentives for subsistence farmers).	
Intersectionality	Meso-social level		
	Infrastructure, health services, social protection services, care services	Reduction and interruptions of essential health services (sexual and reproductive health), childcare (closure of nurseries, schools, and kindergartens), and first response to violence (shelters closing for victims of violence and increased demand for shelter services), need for human resources trained in gender-based violence, limited access to connectivity, errors in public money transfer systems excluding vulnerable people or non-compliance or bad implementation, inefficiency in the operationalization of public policies, poor risk communication.	Promote essential and free remote assistance and support programs (via WhatsApp, email, app, platforms, and 24-h care) for victims of violence to facilitate access to essential services. Analyse the context of connectivity and digital literacy. Increase Internet and communication diapositive’s (cell phones) access, including rural and remote areas. Promote access to health services (for example, sexual and reproductive health or trans people to gender transitioning services), social protection (especially in childcare) and to justice. Promote country guidelines to ensure the availability and access of a minimum package of essential services. Generate clear and transparent protocols for police action and first response services to violence. Provide economic support, direct transfers, unemployment insurance and housing assistance or refugee to survivors of violence. Generate comprehensive services and articulation between essential health, care, social protection and justice programs that take into account intersectionality and accessibility. Promote awareness campaigns and prevention of gender violence, through mass media (television, radio and social networks) with relevant materials from a cultural and local perspective. Generate access to information on protection measures for survivors of violence (as community centres, shelters, sites to report their situation of violence). Support activism, feminist movements, and initiatives of solidarity and mobilization so that governments respond to cases of gender violence. Support social and community organizations in the provision of violence services (although not a substitute for the government and the State). Promote the involvement of civil society in the design, implementation and evaluation of public policies and programs, promoting initiatives originating in the community. Promote gender-sensitive justice and victim response systems with the generation of regulations. Train health, social protection and justice professionals from an intersectional approach. Develop co-interventions (for example, promote information on reporting violence in health centres, public centres, shopping malls, etc.). Generate transversal public initiatives in different areas of gender empowerment that promote parity. Review the performance of the money transfer systems and social protection.
	Civil society	Limited budget to provide services, lack of coordination between government and civil society, the State does not intervene and leaves the work on gender and violence to NGOs.	
	Legislation and access to justice	Laws that are not accessible (included language barriers), discriminatory and with unequal application, barriers to justice, courts, and local and religious counsellors (due to lack of public transportation, service interruptions), hostile judicial institutions not focused on survivors, police abuses, unfair informal and formal justice system, laws influenced by the patriarchal culture (including religion), long judicial processes with bureaucratic logistics, judicial personnel without technical knowledge on protection measures, cases of domestic violence that are not considered emergencies, binary laws that result in violence against a diverse gender.	
Intersectionality	Macro-social level		
	Macroeconomy	Macroeconomic instability, external dependence, recession, indebtedness, economic crisis, inflation of basic products (food), reallocation of funds, increasing levels of austerity, priority to debt service over investments in health, dependence on international financing to support policies of gender.	Assure and increase the financing of community organizations of gender violence with continuity in the provision of services. Generate an efficient allocation of resources in crisis and post-crisis. Promote coordination and federalism within the countries. Generate initiatives, alliances and dialogues between the State, communities, international institutions, civil society, activists, academia, professionals, and local officials. Promote a unified data surveillance and monitoring system with a gender approach from the different sectors (health, social protection, judicial and community), more data and research needed in the area. Work on a comprehensive and intersectional gender strategic action plan that includes not only immediate responses (first response services, childcare, unemployment insurance, economic aid, temporary housing, shelters, complaints, sexual and reproductive health services, food security), but also medium- and long-term responses based on access to stable resources and opportunities (education, employment, income, housing, institutions, structural and cultural changes). Foster recognition of care policies as an integral part of social, economic and employment policies. Generate knowledge from intersectionality and diversity with systematization of local experiences during the pandemic that serve as a starting point for the design of timely policies with a view to recovery. Assess the existing social security and care networks and policies, comprehensively, critically, deeply and detect any possible needs for improvement.
	Public spending for protection	Cut downs on public spending, elimination of subsidies to basic goods, poorly financed gender institutions, support for “masculinized” industries and scarce support for “feminized” ones, lack of funds for childcare centres (or closure of existing ones), little support for care policies, cash transfer programs with values far below social needs.	
	Governance and public policies	Lack of coordination between different policies and fragmented institutions, ideology of the government in office, armed conflicts, little focus on intersectionality in policies (simplistic approaches), gender bias in state organizations, little transparency and low trust of public institutions, lack of health governance, controversies in political discourses, health approach in the emergency that excludes social considerations (such as gender), government scientific advisers without training in gender analysis, problems of implementation of policies and regulations, poorly financed gender ministries, ultra-neoliberal governments, extractive capitalism that exploits and destroys the environment, gender-segregated quarantines, environmental catastrophes.	
	Monitoring, evaluation, and data	Limited data on violence, lack of reliable, complete and scattered data, gender bias in data reporting, and data on gender violence managed by other community institutions are not captured.	
Gender roles			
	Reproductive activities	Exacerbated and additional domestic activities during confinement (household chores, caring for children, elderly and sick), masking of gender divisions in domestic work, mothers leaving the labour force (more than fathers), entrenched beliefs that women are responsible for care, marginalization of reproductive work, increased triple burden work during the crisis (job, domestic and community volunteering demands), interruptions of social connections, less support, lack or disruptions, or conditional eligibility in childcare services, restore of the traditional gender.	Establish care as a public policy problem. Guarantee the opening, operation and full access to the childcare system, kindergartens and schools. Provide additional and targeted support for families seeking to alleviate the increased burden of home care. Promote initiatives so that women can return to work “full time”. Promote initiatives that generate more equality for unpaid activities in households. Generate educational campaigns appealing to the co-responsibility of care, and re-education of men who exercise gender violence and promote new masculinities that challenge the patriarchal culture.
	Patriarchal cultures	Hegemonic masculinities, power asymmetries, patriarchal institutions and societies, social prejudices, during the crisis, the home emerges as a place of subordination, work and violence, women are less likely to make decisions in the crisis without autonomy over sexual and reproductive health.	
Asymmetries	Inequalities		
	Sexism, female leadership, and political discourse	Predominance of the male gender in crisis management (for example, in expert committees) whit low transparency, gender stereotypes in institutional practices, leaders with the use of macho and warlike language, increasing the number of women does not necessarily lead to greater gender awareness in the decisions, prejudice against women, sexist institutional barriers, laws that reinforce the gender-binary, racism, discrimination.	Promote the participation of women and other gender-diverse groups in politics, crisis management and positions of power, considering the gender quota. Promote access to secure sources of information during the crisis. Promote committees sufficiently representative, interdisciplinary and intersectoral (including gender specialists and civil society), considering mechanisms of transparency in decision-making during the crisis and post-crisis. Promote the elimination of sexist institutional barriers by generating equality laws and measures to eliminate sexual and gender discrimination in public, institutional and power spaces. Generate efforts to address and reduce structural inequalities. Promote policies, programs and interventions based on intersectionality integral wellness with the involvement of society. Work on educational instances and awareness addressing gender stereotypes, sexist and macho culture, and promoting the empowerment of women and other groups of diversity. Guarantee compliance with, and access to, regulations on personal data and gender identity. Generate community networks for the approach, prevention and early detection of violence and gender inequalities at the territorial level. Generate the institutionalization of gender equality both at the macro level associated with formal rules and at the micro-level associated with individual perceptions and practices. Support feminist and diversity activism, promoting solidarity and social justice. Establish mental health support channels for diverse groups and generate and promote timely and specific actions. Promote accessing health services for LGBTIQ+ groups, within essential health services and prevention of the LGBTIQ+ phobia in the health institutions and other spaces.
	Gender-based violence	Multiple violence’s (physical, psychological, sexual, economic and social), structural violence, normalization of violence, workplace harassment, domination, sexual exploitation (with a lack of protection systems), women locked up in their homes with their abusers (increased risk of perpetration or intensification of the pre-existing risk with reduced opportunity to seek help), closure of schools leaving girls vulnerable to spending time with potential abusers, presence of problematic substance use, assault, and use of firearms, use of fear of contagion, and control of information such as mechanisms of abuse, increase in femicides carried out by men, prison permits for people for crimes of gender violence during the crisis, presence of double victimization (perpetration of violence and failures of protection systems), difficulties of survivors in accessing the complaint (with closure of public transport), entrenched culture that approves of violence as a means to control and subjugate women and culture of silence, human trafficking for prostitution, drug trafficking, police crackdown, child marriage and forced marriage.	
	Gender discrimination and violations of human rights	Gender-binary segregated restrictions, police brutality to enforce measures, abuses in quarantine centres, increased violence, margination, classism, racism, oppression, discrimination in crisis towards LGBTIQ+ community, sex workers, socially disenfranchised, ethnic minorities, sexual minorities refugees, migrants, or people with disabilities (even with the presence of more than one of these categories) are more likely to experience social problems (for example, food insecurity, violence, housing issues, job loss, lack of state protections or responses that exacerbate disparities), greater difficulty seeking and obtaining help, disclosure of personal data (problematic for some groups, difficulty in accessing protections stated in the gender identity law.	

LBGTIQ+ – lesbian, gay, bisexual, trans, intersex, and queer+, NGO – non-governmental organizations

Cook et al. [26] showed how a short-time work scheme implemented in four European countries with different welfare regimes relied on a normative (male) worker without questioning the gender division of domestic work [26]. Another study carried out in Vietnam also highlighted the limitations of the development and implementation of protection policies to support ethnic minority groups where there has been no gender analysis [78].

Other aspects related to the loss of employment and income were observed, especially for Indian migrant women; their physical presence at the job site was crucial, as their jobs cannot be done online [40]. Moreover, it was observed that black women disproportionately lost jobs in United States [42].

A study in India found a greater loss of employment among women than among men during lockdown [46]. A qualitative study in India, carried out in a group of women from the informal labour sector during the lockdown, revealed how the multiple intersecting forms of inequalities create a complex “matrix of domination” including gender, caste, class, occupational, and religious identities [44].

A study in Bangladesh revealed critical factors related to women's continuing or closing down small businesses during the pandemic in a highly patriarchal context [62].

However, a study carried out in Canada from February to October 2020 showed that gender gaps which existed in employment in the parent group narrowed when restrictions were relaxed [72].

Other aspects at the meso-social level also emerged in the studies, such as reduction of access to services and infrastructure for protection and justice [30,32], or macro-social level markers, such as macroeconomic instability, reduction of public spending for social protection, and different problems on government data monitoring level. Omukuti et al. [21] indicate how financial dependency and austerity and reduced public spending on social services shaped COVID-19 responses in Latin American and Sub-Saharan African countries, showing the importance of recognizing macroeconomic factors as drivers of gender vulnerability in the COVID-19 pandemic.

#### Gender roles

Lockdown and social distancing policies were presented in the studies as strongly related to the dimension of social and cultural roles on reproductive and care tasks linked to women, which were widely present in most studies, though in different depth or scope, or just enunciated (**Table 1**) [19-85].

Reproductive tasks were exacerbated and increased during the pandemic and by the government’s lockdown measures. Additionally, during this period, a triple workload was generated: paid, domestic, and community work [35,47].

As Gordon-Bouvier postulated, there was a crisis of exhaustion and reduced resilience during the pandemic, particularly impacting those engaged in social reproduction, both inside and outside the home [73].

Moreover, a study in Australia found that the rise in “relative equity” during the lockdown did not compensate for the extra unpaid work burden the pandemic caused for women [41]. These measures also had negative impacts on the scholarly productivity in the group of female academics [24,60].

A study in Panama also delved into gender-segregated distancing policies, indicating lower visits to all community location categories on female-mobility days. As the authors claim, women could have undertaken fewer tasks outside the home than men [65].

#### Asymmetries and inequalities

The implemented policies related to the dimension of asymmetries and inequalities; although they were stated in several texts, they took on a central role in studies specifically addressing gender-based violence (GBV) during the lockdown period (**Table 1**) [22,27,30,32,36,39,43,50,51,58,61,69,70,75,76]. Difficulties in accessing essential services by survivors of GBV, both because they were unable to travel or seek help and because services were reduced during this period, were also reported in these studies [22,27,30,32,43,58,76].

Survivors of GBV with marginalized identities have been at greater risk of being doubly victimized by the perpetration of violence and by the failure of protection systems [22].

Dias Corrêa et al. [50] highlight that structural violence worsened during the pandemic lack of care by of the State.

Also, as indicated by Srivatsa [57], communication and outreach by local governments is critical. John et al. [43] showed how the Kenya government turned its attention to GBV only after reports of rising GBV led to advocacy by activist.

In this dimension, asymmetries related to the low presence of women in pandemic management committees or decision-making positions during the crisis were also visualized [23,25,29].

A study conducted in Canada and Scotland by Soremi et al. [35] indicated that female political leaders do not need to base their legitimacy on gender, even more so in environments where these policies have already been institutionalized, adding that the emphasis should be on professional progress.

In their global study, van Daalen et al. [23] showed how women's voices were excluded in expert working groups and decision-making during the pandemic, with very low gender parity. Furthermore, van Daalen et al. [23], Bacigalupe et al. (in Spain) [29], and Sell et al. (in Germany) [48] found a deficient level of transparency on the committees’ composition.

Wenham et al. [25] found that, although the average number of women in scientific advisory groups on emergencies increased during the crisis, this did not imply a greater awareness of gender issues in politics. Similar ideas were expressed in other studies [20,23].

Kim et al. (in South Korea) [20] indicate that COVID-19 mitigation policies were sustained through a masculinized discourse related to policies focused on minimizing the spread of the virus and alienation from other social problems.

Perez-Brume et al. [36] clearly show the presence of violence and marginalization generated during the pandemic, pointing out serious and unacceptable situations suffered by transgender groups in some Latin American countries, such as Peru, Panamá, and Colombia, where the physical distancing policy was based on binary interpretations of gender.

The Irons’ study analysing gender-segregated quarantine in Peru suggests that these events were more than just the results of the policy-makers’ missteps, but rather the persistence and exacerbation of long-existing of colonial and patriarchal structures [52].

Rieger et al. [22] enunciated elements related to gender diversity, indicating that most survivors of gender-based violence are women and gender-diverse people. Different studies in Brazil, India, and Indonesia indicated psychological distress or mental health problems in gender-diverse persons during the pandemic, social distancing, and lockdown periods [49,61,67,70].

The pandemic’s consequences for diversity groups are varied, with an increase in inequities and LGTBI-phobia presence [49]. Studies in Brazil and India [49,61,70] have shown that it exacerbated stigmatization and marginalization in already marginalized groups [61] such as gender-diverse persons.

However, Rodriguez Fernandez [34] and Polischuk et al. [39] provide examples of violence prevention services or social programs during the pandemic that included members of the LGBTIQ+ communities, as was the case of Argentina and New Zealand.

Also, the negative consequences of the closure were also observed in other groups such as sex workers, exposing them to exploitation by both their clients and the police [43]. It was also detected in India, where the patriarchal structure and social prejudices conditioned women's experiences of the COVID-19 crisis [44]. Studies have also pointed out the humiliation of migrant workers from India by state authorities during lockdown [45]. Dias Corrêa et al. [50] and Camilo et al. [51] also pointed out the structural violence from the police force in Brazil.

A study analysing the case of India indicated how the state and social mechanisms of power, following the pandemic outbreak, pushed the populations into precarious living situations and conferred upon them the status of “living-dead” [45].

Using the content analysis of the included documents (**Table 1**) [19-85] and the emerging framework of socio-cultural markers (**Figure 2**), we developed a matrix to identify and adapt the main challenges and recommendations detected for consideration during the pandemic (**Table 2**).

## DISCUSSION

The disease caused by COVID-19 was first reported in Wuhan, China in December 2019 [86]. To date (May 11, 2022), the World Health Organization has reported 516 476 402 confirmed cases and 6 258 023 deaths globally [87].

The COVID-19 pandemic has triggered unprecedented governmental and political action worldwide [88]. Strict health measures such as social distancing policies, isolation, and lockdown, have been implemented in many countries [89] and were the focus of many studies included in this review.

Although these measures are effective and imperative in curbing the spread of infectious diseases [89-91], they have also had negative effects on multiple spheres of wellness, and they are the subject of analysis due to their economic, social, and psychological repercussions [89-92].

The pandemic has amplified multiple existing inequalities [92], especially those related to gender as found in this study, but it also occurred at a time of demands for social change and greater equality with a growing feminist movement from before the onset of the COVID-19 pandemic [21]. However, the public health policies promoted during the crisis have not addressed the gendered impacts [2]; this has occurred in previous disease outbreaks [2,31,93] and was amply demonstrated in this study.

This omission of gender in health policies during the pandemic, in part, can be explained by the “tyranny of the urgent” [93,94], marked by a dissociation between immediate biomedical needs and those not considered a priority, such as inequities and structural problems.

This study also highlighted the methodological and scope limitations of studies published during this stage. A predominantly binary approach to gender has been observed, similar to what Williams et al. suggest [5].

Gaps were also evident at the level of the policies analysed and their impacts, as proposed by Agarwal [95]. The literature was largely focused on the immediate confinement measures and the consequences on care/domestic work and violence [95], while there was no in-depth and central analysis of other inequity markers related to medium and long-term impacts that will surely affect recovery, such as food and nutritional insecurity, loss of livelihood, indebtedness, low resilience, and rising poverty [95]. Food and nutritional insecurity issues were considered by Chitando [31], Oladeinde [33], Arora et al. [40], Singh et al. [44], Bau et al. [55], and Pinchoff et al. [69] carried out in African countries and India. A part of the documents analysed the gender impacts globally and immediate employment consequences. The limitation in the scope of gender markers in the analysed studies was probably since most were conducted during the early stage of the pandemic, raising the need to generate new visionary and localized evidence.

Smith [93] postulated that, while health policy research may have incorporated gender analysis, few specific studies on gender issues are related to outbreaks. We found that gender gaps are still noticeable, as was reported in previous studies [93,95].

Another aspect to be highlighted was the presence of a smaller number of documents in some regions, such as Africa, despite the evidence of the COVID-19 pandemic’s substantial impact and the partial and inconsistent policy response with respect to gender in this continent, as in Latin America [21]. Low- and middle-income countries show problems of gender inequity with fragile health systems; they should be more proactive in improving their evidence-based strategies to provide sustainable solutions and reduce the different gaps [96], requiring more studies in this territory.

Despite the limitations of the literature, a multiplicity of socio-cultural markers was found that translate into present and future gender-related challenges caused by the pandemic and the measures implemented by governments.

This study sought to address the policy measures generated during the crisis from an intersectional and located approach, not only considering individual conditions but a broader framework that recognizes the social and geopolitical forces that shape people's lives [97], assuming that communities are not homogeneous and that there is a diversity of experiences [45,97], and that the impacts of COVID-19 and the implemented policies will also be differential.

As we have observed, people with one or more identities (such as marginalized people, disability people, undocumented people, ethnic minorities, people of colour, sexual and gender minorities, migrant women, and cisgender women) may be particularly and disproportionately affected by the COVID-19 pandemic [22,97,98].

The gender-blind planning and decision-making in public health during the response to the COVID-19 pandemic stem from a hegemonic and patriarchal system, generating differential needs in these groups [98].

As found in this study, the measures implemented during the COVID-19 pandemic, especially lockdowns and restrictions and have substantially increased the risk of gender-based violence [22,27,30,32,36,39,43,50,51,58,61,69,70,75,76]. Gender and sexual minorities have been severely affected [36,49,56,61,67,70].

Besides the challenges mentioned above, there are other gender-blind spots, which also deserve attention, such as the low opportunities for female decision-making during the crisis [23,25,29,48]. However, the gender analytical lens found focused on the increased risk of gender violence, the domestic and reproductive work, and social inequalities, especially in employment and income [19-85].

This study’s main strength is the comprehensive approach to the gender approach at both the policy and knowledge levels generated during the COVID-19 crisis.

Further research in this field will be necessary to reduce the current knowledge gap; to our knowledge, there are no published works similar to this study. The global evidence regarding the gender approach in public policies promoted during the COVID-19 pandemic in different countries and contexts until now has never been, to our knowledge, fully synthesized. Another strength lies in the potential of the emerging framework and policy recommendations generated from the data, which could serve, although duly adjusted and adapted, to post-pandemic contexts and future health, humanitarian, and environmental crises.

Limitations include those arising from the design itself and its descriptive and exploratory nature. However, the purpose of this work was not to systematically compare the studies, but rather to describe and comprehensively discuss the “state of the art” on the subject from a theoretical, methodological, and analytical point of view in order to generate policy recommendations and more specific future research.

Due to the nature of the study, we have not evaluated the quality of the evidence and there may have been information that was not captured and included by the search. For pragmatic and feasibility reasons, it was necessary to limit the search strategies in three languages (although the language was not an exclusion criterion) and during the initial screening stage, only one researcher reviewed the abstracts and titles. The review may have missed documents written in other languages.

However, we have made different efforts to achieve the revision of an important diversity of databases that allows us to capture the greatest possible number of experiences from different continents and countries.

A series of measures were taken to minimize inclusion biases: a researcher reviewed the titles and abstracts of all records and all full texts at least twice. A sample of studies excluded in the abstract and title review again underwent a full-text review, and three researchers independently reviewed the potentially included studies.

Publication bias could have existed, considering the emergency context (as much of the work was generated during the first months of the pandemic), making it less likely for studies with positive results or implementation of gender protection policies to be published. However, the focus of this work was based on inequities, although studies from countries where positive strategies were discussed.

Other limitations were the gender imputation of the first author through information collected from the web rather than from self-perception and that the historical, ideological, political, and normative differential context of each country with respect to gender advances was not considered. However, a certain consistency was found among the analysed studies regarding the approach and scope, which may indicate that, despite context-specific gender norms, the effects of the pandemic and the implemented policies could transcend geographical barriers, languages, social and cultural contexts, similar to those reported in a previous study [25].

## CONCLUSIONS

This study highlighted the multiple gender gaps in both knowledge generation and policy implementation during the COVID-19 pandemic in different countries. The lockdown policies negatively affected multiple dimensions of wellness for women, socially vulnerable groups, and other diverse identities.

This should be considered a call to action for researchers, policy-makers, and other stakeholders to incorporate a gender-diverse perspective into the policy field in an intersectional sense to overcome current inequities and asymmetries and not perpetuate and deepen them.

The COVID-19 pandemic represents a unique opportunity to realign policy priorities from inclusive and gender-transformative approaches to recovery.

## Additional material:


Online Supplementary Document
