# Supplementary material for: Gender and social protection and health policies promoted during the COVID-19 pandemic: Global scoping review and future challenges
Source: J Glob Health. 2022 Dec 29;12:05056. doi: 10.7189/jogh.12.05056 (PMC9798348; doi:10.7189/jogh.12.05056)
Supplement: Online Supplementary Document [file jogh-12-05056-s001.pdf]

ONLINE SUPPLEMENTARY DOCUMENT

Title

Gender and social protection and health policies promoted during the COVID-19 pandemic: global scoping review and future challenges

Authors

Daniela Luz Moyano, PhD
María Lara Martínez, PhD
Laura Lara Martínez, PhD

Appendix S1: Search strategies

MEDLINE (English)**:** (Policy[Mesh] OR Public Policy*[tiab]) OR Health policy*[tiab]) AND (Sex[Mesh] OR Sex*[tiab] OR Gender Identity[Mesh] OR Gender Identity[tiab] OR Gender*[tiab] OR Health Disparity, Minority and Vulnerable Populations [tiab]) OR Sociological Factors [tiab]) OR Social Problems [tiab] OR Social Discrimnation [tiab] OR Social Behavior [tiab]) AND (COVID-19[Mesh]).

Anthropological Index Online (English, Spanish and Portuguese): “Gender”, “COVID-19”. Género, “COVID-19”. Gênero “COVID-19” (In keywords).

Google Scholar (English, Spanish and Portuguese): "Gender COVID 19 Policy"; "Gender COVID 19 Policies". "Género COVID 19 Política"; " Género COVID 19 Políticas". "Gênero COVID 19 Política "; " Gênero COVID 19 Políticas" (With restriction of presence in the title of the article).

JSTOR Journal Storage (English): “Gender” “COVID-19” “Policy” (Title includes gender and COVID-19, and in all fields only policy).

SAGE Journals (English): “Gender” “COVID-19” “Policy” (In keywords).

EMBASE (English): "gender" "COVID-19" "policy" (In title, abstract, and keywords).

Springer (English): "Gender AND COVID-19" “Policy” (gender and COVID-19 as exact phrases, and in all Policy fields).

Studies on Women & Gender Abstracts (English): "COVID-19" AND "policy" AND "Gender" (In title, abstract, and keyword).

Cochrane Library (English): “gender” AND "policy" AND “COVID-19” (In title, abstract, and keyword)

Global Index Medicus (included regional indexes: African Index Medicus–AIM, Latin America and the Caribbean Literature on Health Sciences–LILACS, Index Medicus for the Eastern Mediterranean Region–IMEMR, Index Medicus for South-East Asia Region–IMSEAR, Western Pacific Region Index Medicus–WPRO) (English): “gender” AND "policy" AND “COVID 19” (In title, abstract, and keyword).

In Lilacs (English, Spanish and Portuguese): Policy AND (Gender) AND (COVID-19). Política AND (Género) AND (COVID-19). Política AND (Gênero) AND (COVID-19) (In search criteria).
